# Fracture Behavior of Permeable Asphalt Mixtures with Steel Slag under Low Temperature Based on Acoustic Emission Technique

**DOI:** 10.3390/s20185090

**Published:** 2020-09-07

**Authors:** Bing Zhu, Hanbing Liu, Wenjun Li, Chunli Wu, Chao Chai

**Affiliations:** College of Transportation, Jilin University, Changchun 130025, China; zhubing18@mails.jlu.edu.cn (B.Z.); lhb@jlu.edu.cn (H.L.); wenjun18@mails.jlu.edu.cn (W.L.); chaichao18@mails.jlu.edu.cn (C.C.)

**Keywords:** fracture behavior, permeable asphalt mixtures, steel slag, low temperature, acoustic emission

## Abstract

Acoustic emission (AE), as a nondestructive testing (NDT) and real-time monitoring technique, could characterize the damage evolution and fracture behavior of materials. The primary objective of this paper was to investigate the improvement mechanism of steel slag on the low-temperature fracture behavior of permeable asphalt mixtures (PAM). Firstly, steel slag coarse aggregates were used to replace basalt coarse aggregates with equal volume at different levels (0%, 25%, 50%, 75%, and 100%). Then, the low-temperature splitting test with slow loading was used to obtain steady crack growth, and the crack initiation and propagation of specimens were monitored by AE technique in real time. From the low-temperature splitting test results, SS-100 (permeable asphalt mixtures with 100% steel slag) has the optimal low-temperature cracking resistance. Therefore, the difference of fracture behavior between the control group (permeable asphalt mixtures without steel slag) and SS-100 was mainly discussed. From the AE test results, a slight bottom-up trend of sentinel function was founded in the 0.6–0.9 displacement level for SS-100, which is different from the control group. Furthermore, the fracture stages of the control group and SS-100 could be divided based on cumulative RA and cumulative AF curves. The incorporation of 100% steel slag reduced the shear events and restrained the growth of shear cracking of the specimen in the macro-crack stage. Finally, the considerable drops of three kinds of *b*-values in the final phase were found in the control group, but significant repeated fluctuations in SS-100. In short, the fracture behavior of PAM under low temperature was significantly improved after adding 100% steel slag.

## 1. Introduction

Permeable asphalt pavement refers to the fully permeable pavement in which rainwater permeates into the ground through subgrade or discharges through buried drainage facilities [1]. Porous asphalt pavement refers to the semipermeable pavement in which rainwater penetrates into the surface course and is horizontally drained by buried pipelines. However, referring to the literature in recent years, the difference between them is not obvious [2]. Permeable asphalt mixtures (PAM), as sustainable pavement materials, are credited with various environmental benefits, such as urban floods alleviation by promoting the natural infiltration of rainwater, underground water quality improvement by treating pollutants, and heat-island effect mitigation by facilitating heat transfer processes [3,4]. However, their poor low-temperature cracking resistance has become a barrier against the wide application of PAM [5,6].

Steel slag is the main byproduct of the steel manufacturing industry [7]. According to the data from the World Steel Association, the crude steel production output of China in 2019 has reached 996.3 million tons [8], but the utilization rate of steel slag in China is only 29.5% [9]. A large number of steel slags have been disposed of as waste, leading to a series of serious social and environmental problems, such as the occupation of limited land, heavy metal pollution of groundwater, and dust pollution of the air [10,11]. Due to the excessive exploitation of natural aggregate, limited natural aggregates cannot satisfy the increasing demand for road construction [12,13]. Therefore, it is urgent to find a high-quality and low-cost alternative for natural aggregate [14]. The mechanical performance of PAM is poor and can be enhanced by high-quality aggregates [15]. Fortunately, steel slags can be served as an attractive substitute for natural aggregates due to its superior characteristics including low cost, high wear resistance, toughness, multi-angularity, and good adhesion with bitumen, compared with other aggregate substitutes [16,17].

Owing to the superior characteristics of steel slag, its application in PAM has received extensive attention in recent years. Skaf et al. [18] carried out a series of tests to examine the effect of steel slag on the pavement performance of PAM, including mechanical performance (abrasion loss and indirect tensile strength), durability (cold abrasion loss, aging, and long-term behavior), water stability, skid and rutting resistance, and permeability. The results showed that the addition of steel slag significantly improves the permeability, skid resistance, durability, and rutting resistance of PAM. Rodríguez-Fernández et al. [19] and Chen et al. [20] found that steel slag can improve the economy and environmental sustainability of PAM by life-cycle cost analysis (LCCA). Existing research studies about the low-temperature behavior of PAM focused on natural aggregate, but few focused on steel slag [21,22]. Therefore, the effect of steel slag on the low-temperature behavior of PAM needs to be studied. The low-temperature fracture behavior of PAM with steel slag was complicated, owing to its non-uniformly graded skeleton and interlocking structure. Therefore, a simple, feasible, and real-time fracture monitoring technique is needed.

Acoustic emission (AE), as a nondestructive testing (NDT) and real-time monitoring technique, has great potential for the identification of failure stage, the classification of cracks, and the location of cracking sources [23,24,25]. AE is a phenomenon that generates transient elastic waves by the rapid releasing of internal energy from the localized source or sources of materials, which is related to the local irreversible changes, including plastic deformation, crack initiation and propagation, etc. [26,27]. AE technique has been used extensively for damage assessment and health monitoring in diverse fields, including metal, rock, concrete, and bridge, etc. [28,29]. However, relatively limited research works have focused on the damage assessment of asphalt materials using the AE technique. Behnia et al. [30] proposed an AE-based method to determine the embrittlement temperature (*T_EMB_*) of asphalt binders. The results showed that the amount of pavement cracks is proportional to the difference between *T_EMB_* and performance grade (PG) low temperature of asphalt binders. Wei et al. [31] utilized the AE parameters, including amplitude, ringing count, AE energy, and average frequency, to divide the failure stage of asphalt materials under low temperature. The spatial evolution process of internal micro-cracks was tracked by the AE localization technique. Qiu et al. [32,33] utilized AE energy and *b*-value to monitor and evaluate the fatigue damage behavior of asphalt mixtures. Then, the fractal dimension of the AE waveform after wavelet transform was used to identify the damage critical state. The results revealed that the energy distribution of AE waveform can be used to characterize the irreversible damage mechanism associated with Kaiser and the Felicity effects. Sun et al. [34] studied the relationship between AE parameters and oxidation aging performance of asphalt mixture. The existing research mainly focused on the application of the AE technique in dense asphalt mixture (DGM), but it was rarely used in PAM. The characterization of the cracking mechanism of PAM is a complicated and challenging subject [35,36]. Nowadays, some studies demonstrated the feasibility of the AE technique in PAM with heterogeneity and stone-on-stone interlocked structure, and reported that AE possesses great potential in real-time crack monitoring and fracture characterization of PAM, especially in complicated environments. Jiao et al. [37,38] investigated the damage evolution and fracture modes of PAM under low-temperature splitting with the aid of the AE technique. Cai et al. [39] found that the incorporation of lignin fiber can significantly delay the growth of shear cracks along the interface under uniaxial compression by employing the AE technique, and stated that AE can effectively compensate for the deficiency of mechanical experiments and theoretical models. Chai et al. [40] carried out low-temperature splitting and AE tests on the specimens with different freeze-thaw cycles. The results showed that AE parameters can effectively identify the damage stage of PAM under freeze-thaw cycles. Therefore, the previous research provided experience and guidance for the application of the AE technique in PAM pavement.

However, the low-temperature fracture behavior of PAM with steel slag based on the AE technique has not been discussed in existing research, and especially the *b*-value analysis has not been involved. Therefore, the principal purpose of this paper was to investigate the improvement mechanism of steel slag on the low-temperature fracture behavior of PAM. Steel slag replacement level was designed from 0% to 100% (25% increment). Low-temperature splitting test with slow loading (1 mm/min) was used to obtain steady and slow crack growth, which can be detected employing the AE technique. Firstly, the relationships between mechanical characteristics and AE activities for each group were described by the sentry function. Then, the shear and tensile modes were characterized by the relationship between the rise angle (RA) and average frequency (AF). Finally, the relative variations of micro-cracks and macro-cracks were identified by the *b*-values. The results obtained in this study provided a basis for the superior low-temperature cracking resistance of permeable asphalt mixtures with 100% steel slag (SS-100).

## 2. Materials and Methods

### 2.1. Raw Materials and Mixture Design

Compared with other modifiers, the styrene–butadiene–styrene (SBS) modifier has the optimal comprehensive performance, including excellent modification effect and attractive economic efficiency [41,42]. Therefore, the SBS modified bitumen, produced in Liaoning Province, was used as binder and its basic properties were shown in Table 1. The coarse aggregates used were basalt and steel slag, both from Jilin City, Jilin Province. Their basic properties were shown in Table 2. To eliminate the difference of aggregate specific gravity, steel slag coarse aggregates were used to replace basalt coarse aggregates with equal volume at different replacement levels (from 0% to 100%, 25% increment), marked as the control, SS-25, SS-50, SS-75, and SS-100 groups, respectively. Due to the high expansion and uncleanness of steel slag fine aggregates, only basalt fine aggregates were selected as fine aggregates, and its basic properties were shown in Table 3. Based on the technical specification for permeable asphalt pavement (CJJ/T 190–2012), the target volume of air voids (VV) of the mixture was 20% and the experimental gradation was determined and shown in Table 4 [43]. Based on Cantabro and draindown tests, the optimal bitumen contents (OBCs) of each group were determined as 3.66%, 3.57%, 3.47%, 3.47%, and 3.38%, respectively.

### 2.2. Specimen Preparation

The specimen preparation steps were listed as follows:

- Step1: Place the weighed aggregates and mineral powder in the oven at 180 °C for 6 h and heat the SBS-modified bitumen to 180 °C to reach the viscosity required for mixing.
- Step2: Place the preheated aggregates in a constant temperature mixing pot and stir for 90 s. Then, add the weighed SBS-modified bitumen and stir for another 90 s. Finally, add the preheated filler to mixing for 90 s.
- Step3: Use a standard Marshall hammer to compact the specimens with 50 blows per side.
- Step4: Demold the specimens after 12 h.

For volume characteristics and Marshall tests, there were four identical cylindrical specimens (diameter, 101.6 ± 0.2 mm; height, 63.5 ± 1.3 mm) for each group. For Cantabro, draindown, indirect tensile, and water stability tests, three parallel specimens in each group were used. There were three specimens for each group to carry out low-temperature splitting and AE tests. For the time-independent cumulative index (fracture energy and cumulative AE energy), the average value of three parallel specimens was taken. For the time-dependent process indicators (RA, AF, and *b*-value), if the AE process parameters of the three specimens show a consistent trend, the AE signal of the specimen corresponding to the median cumulative AE energy selected; otherwise, one specimen is added until the trend is consistent.

### 2.3. Volume Characteristics and Mechanical Properties

The volume characteristics and mechanical properties test results of each group were shown in Table 5 and the low-temperature splitting test results were shown in Table 6. The corresponding standard deviation of test results were listed in Table 7. For volume characteristics, it can be found that VV and permeability increase with the increase of replacement level with steel slag, which is consistent with the results obtained by Skaf et al. [18]. In addition, the Cantabro abrasion loss and draindown both reached the minimum (7.8% and 0.16%) at 100% replacement level, indicating that the addition of steel slag can significantly improve the resistance to particle loss and the adsorption capacity of free bitumen. From the Marshall stability (MS), indirect tensile strength (ITS), and tensile strength ratio (TSR), it can be seen that the high-temperature performance, mechanical strength, and water stability are also significantly enhanced, which is consistent with the results obtained by Skaf et al. and Rodríguez-Fernández et al. [18,19]. From the low-temperature splitting test results, it can be found that the incorporation of steel slag has no significant effect on the low-temperature indirect tensile strength (LITS), but the failure strain and fracture energy were significantly enhanced, indicating that low-temperature cracking resistance of PAM is significantly improved. Therefore, the PAM with 100% steel slag has the optimal volume characteristics and mechanical properties. In addition, it can be found that the basic performance of SS-100 meets the specification requirements.

### 2.4. Acoustic Emission (AE) Test

#### 2.4.1. Setup

In our previous study, the feasibility of AE technology in splitting test has been demonstrated [37,39]. In order to ensure the consistency of the tests, we used splitting test to obtain crack propagation. The universal testing machine, with a maximum load of 100 kN, composed of a loading system, control system, and an environmental chamber, was employed to carry out the low-temperature splitting test. Slow displacement control (1 mm/min) was applied to obtain slow and stable crack growth. Before the experiment, the specimens were kept at a constant temperature of −10 °C for 6 h to ensure its internal temperature uniformity. The fracture energy was calculated by integrating the modified load–displacement curve until the maximum load was achieved. This indicator was adopted to evaluate the low-temperature cracking resistance of mixtures, because fracture energy contains more information than failure stress and strain.

**Figure 1:**
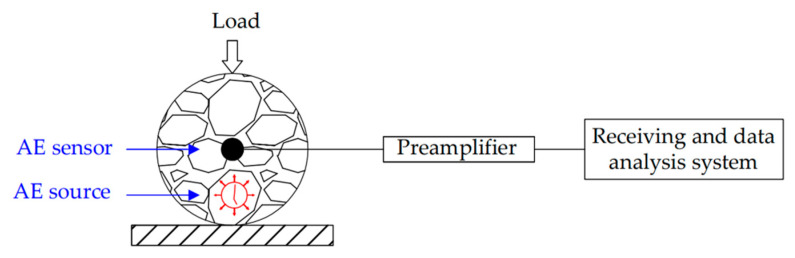
The schematic representation of AE monitoring.

**Figure 2:**
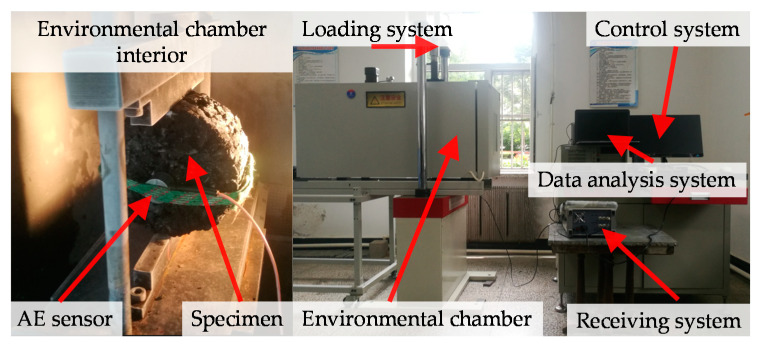
The experimental setup and detailed description for the AE tests.

During the low-temperature splitting test, the AE signals of the sample were recorded in real time by employing the AE monitoring (as illustrated in Figure 1), which consists of a sensor, preamplifier, receiving system, and data analysis system. Firstly, the SR150M 150 kHz resonant sensor with sensitivity within the frequency band of 60–400 kHz was tightly attached to the side of the specimen with an elastic belt. Only one sensor was used, owing to its small specimen size. Additionally, the phenomena of accumulated signal distortion and attenuation caused by the long-distance propagation between the cracking source and the signal receiver can be weakened by employing small-scale specimens (diameter: 101.6 mm; height: 63.5 mm) [44]. 706 silica gel was used as a coupling agent to eliminate the influence of interface air on the signals. Then, the preamplifier gain was set as 40 dB. A six channel SAEU2S data receiving system and data analysis system was manufactured by Shenghuaxingye Technology Co., Ltd., Beijing, China. The threshold was set as 40 dB to reject the environment and mechanical noise. The sampling frequency was 5 MSPS for waveform recording. The peak definition time, hit definition time, and hit lock time were set as 50 μs, 100 μs, and 300 μs, respectively. Finally, the pencil lead breaking test was utilized to calibrate the sensor, in order to check the sensitivity and coupling of the sensor. The aforementioned steps ensured the integrity and accuracy of AE signals. The experimental setup and detailed description for the AE tests were shown in Figure 2.

#### 2.4.2. AE Basic Parameters

**Figure 3:**
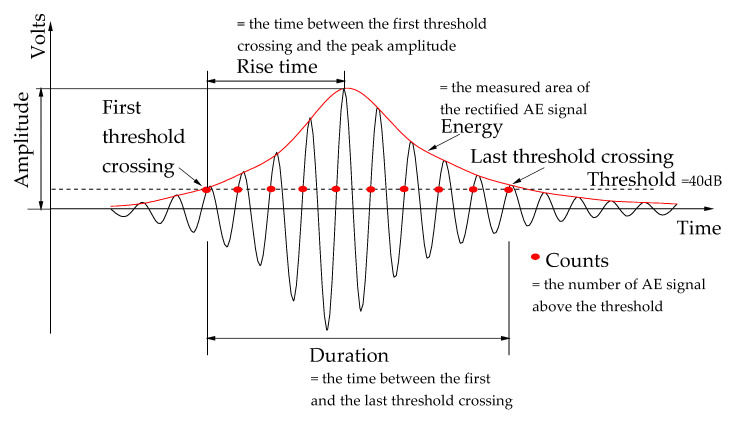
AE basic parameters.

The AE basic parameters, namely amplitude, rise time, duration, counts, and AE energy, were illustrated in Figure 3.

The AE energy and cumulative AE energy can be calculated by:

(1) $$E_{AE}=\int_{0}^{t}V^{2}\left(t\right)dt$$

(2) $$CE_{AE}=\sum_{t=0}^{t=t_{i}}E_{AE}\left(t\right)$$

where *E_AE_* is the AE energy (mv·ms); *CE_AE_* is the cumulative AE energy (mv·ms); and *V*(*t*) is the recorded voltage at time *t*.

The AE energy was more sensitive to identify the damage evolution than other basic AE parameters, owing to the fact that it contained more signal source information [37]. Additionally, the AE energy was related to crack relative intensity and severity [45]. The greater the AE energy, the greater the crack intensity, and vice versa. To distinguish the intensity of cracks, the signals with AE energy lower than 0.5 mv·ms and higher than 1.0 mv·ms were defined as low-energy signals and high-energy signals, respectively, and the signals between them were called medium-energy signals.

The sentry function was an index that simultaneously considered both load-displacement curve and cumulative AE energy curve. It was used to describe the relationship between mechanical characteristics and AE activities of material [46]. Thus, it was superior to the AE basic parameters contained single information [47]. It was defined as the logarithm of the ratio between the stored mechanical energy and the released AE energy, and given by the following equation:

(3) $$f\left(x\right)=\ln\left(\frac{E_{M}\left(x\right)}{CE_{AE}\left(x\right)}\right)$$

where *x* is the displacement level; *f*(*x*) is the sentry function at *x* displacement level; *E_M_*(*x*) is mechanical energy at *x* displacement level calculated from the modified load-displacement curve, in joules; and *CE_AE_*(*x*) is the cumulative AE energy at *x* displacement level as mentioned above.

Depending on the failure process of materials, the sentry function illustrates five different trends during its loading history [48,49]. Therefore, it is related to different damage phases of materials. The strain energy storing phase is characterized by an upward trend of sentry function in the early stage. The reason is that a small amount of AE signals are excited, while the elastic energy is gradually accumulated in the early stage. The damage accumulation phase is characterized by a steady downward trend of sentry function, which is associated with the instantaneous release of stored strain energy generated by the internal damage of materials. The steady phase is described by constant behavior of sentry function, which is associated with the semi-balance state between the internal damage such as cracking growth and hardening effects such as fiber bridging and self-healing effects. The hardening phase is characterized by a subsequent bottom-up trend of sentry function, indicating that the hardening effect is stronger than the internal damage in the local region. When the sentry function is at a low value and dropped sharply, the mechanical properties of the material is deteriorated significantly and the AE activity is remarkable in the failure stage.

#### 2.4.3. Rise Angle (RA) and Average Frequency (AF)

Rise angle (RA) and average frequency (AF), as well-known features, have been proved to be the key parameters to characterize the tensile and shear modes [38]. *RA* and *AF* are obtained from the AE basic parameters, which can be expressed as follows:

(4) RA=Rise time/Amplitude, ms/v

(5) AF=Counts/Duration, kHz

The unit of amplitude directly obtained from the AE signal is decibels. However, it can be found that the unit of amplitude in this case is volts, and the conversion formula is as follows [50]:

(6) $$A_{dB}=20\log\left(\frac{V_{\max}}{V_{ref}}\right)$$

where *A_dB_* is the amplitude measured in decibels (dB); *V*_max_ is the largest voltage peak (also called the amplitude expressed in volts); and *V_ref_* is the reference voltage (*V*), which is generally 1 μV (voltage generated by 1 mbar pressure).

**Figure 4:**
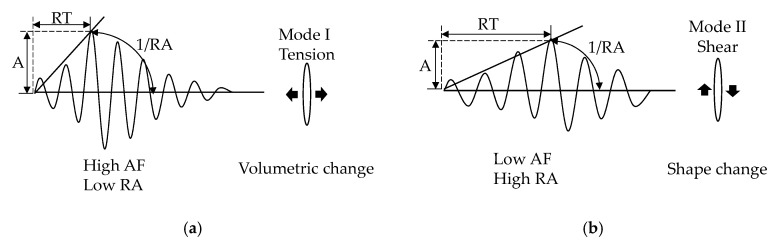
Signal waveform diagram and corresponding cracking modes: (**a**) tensile crack; (**b**) shear crack.

When the specimens are subjected to external loads, some fracture modes, including shear cracks and tensile cracks, occur. The shapes of AE waveforms are associated with the fracture modes of shear and tensile cracks [32]. The tensile cracks can excite energy in the form of longitudinal wave and cause the volume change of material. Differently, when the shear crack occurs, the elastic energy release in the transverse wave mode and the shape change of material happen. Compared with the shear wave modes, the tensile wave modes have a shorter rise time and duration time, as well as higher amplitude and counts, resulting in higher AF and lower RA. Conversely, shear wave modes have a longer rise time and duration time, exhibiting higher RA and lower AF values, because the shear wave has a slow propagation velocity and takes a longer time to reach its peak in the process of its formation and propagation [44]. The typical signal waveforms of tensile crack and shear crack were shown in Figure 4.

**Figure 5:**
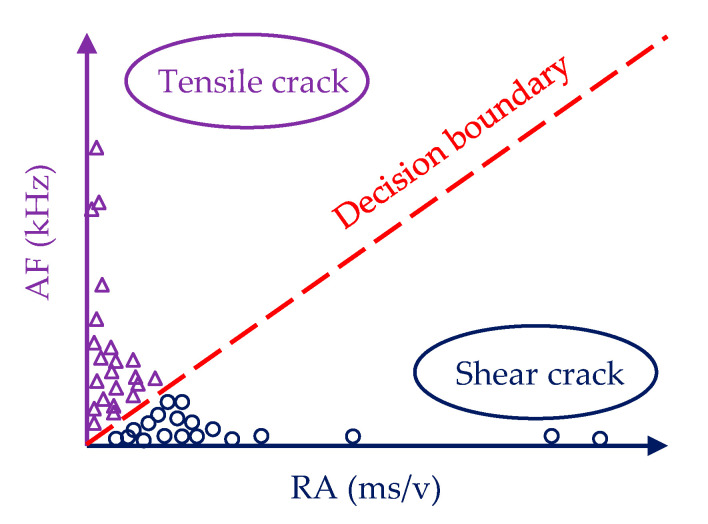
Classification line.

According to Japanese building code JCMS–III B5706, a conventional and simple classification criterion that a straight line (illustrated in Figure 5) passing through the origin was adopted to categorize tensile and shear crack in this study [51]. However, the slope of the general boundary line has not been given owing to the differences in geometry, structure, and material of the specimens. In this paper, a new method, similar to the *k*-medoids clustering algorithm, was proposed to determine the slope [38]. The objective function was described as the dissimilarity of all objects in the clusters and calculated by giving the specific slopes (from 0.1 to 1, 0.1 increment; from 1.0 to 10.0, 1.0 increment). The optimal value of the slope was determined based on the minimum dissimilarity. The objective function could be expressed as:

(7) $$F=\sum_{j=1}^{2}\sum_{x_{ij}\in cluster:j}{d_{E}}^{2}\left(x_{ij},c_{j}\right)$$

(8) $${d_{E}}^{2}\left(x_{ij},c_{j}\right)={\left(x_{ij}-c_{j}\right)}^{T}\left(x_{ij}-c_{j}\right)$$

where *F* is the objective function; *j* is the cluster (*j* = 1 or 2, representing shear crack and tensile crack, respectively); *x_ij_* is signal points belonging to *j* cluster (*I* = 1, 2, …, n); *c_j_* is the centroids of cluster *j*; and the *d_E_*^2^ (*x_ij_, c_j_*) is the squared Euclidean distances between the *x_ij_* and the *c_j_*.

#### 2.4.4. *b*-Value

The *b*-value has been proved as a good indicator to monitor the progressive damage evolution and capture the precursory information about structural collapse, owing to the schematically and regular changes of the *b*-value during the loading period [52]. The high *b*-values indicated a large number of events with low amplitude, associated with the initiation and propagation of micro-cracks in the early stage. In contrast, the low *b*-values meant that a small number of events with high amplitude occur since those events mainly originate from the formation and unstable propagation of macro-cracks in the terminal stage. Thus, *b*-value was used to identify the relative variation of micro-cracks and macro-cracks. In order to guarantee the accuracy and integrity of AE data analysis, amplitude *b*-value based on the least square method (*b_L_*-value), amplitude *b*-value based on the maximum likelihood method (*b_M_*-value), and energy *b*-value (*b_E_*-value) were all taken into consideration.

The traditional *b*-value was defined as the negative logarithmic gradient of the frequency-magnitude distribution by employing the empirical Gutenberg-Richter law, which has been widely applied in seismology to characterize distributions of earthquake magnitude [53]. The formula is as follows:

(9) $$Log_{10}N\left(\geq M\right)=a-bM,$$

where *M* is the magnitude of seismic wave events on the Richter scale; *N*(*≥M*) is the cumulative number of events with magnitude greater than *M* in a given region and specific time range; *a* and *b* (also called *b*-value) are the intercept and the negative slope of the regression line based on the least square method in seismology, respectively.

There are similarities between the AE phenomena in the process of structural fracture and the seismic activities on the region of the Earth’s crust, owing to the size independence of the *b*-value [44]. Application of the Gutenberg-Richter law to seismic waves was extended to the AE technique field in a small-scale way after dividing the AE amplitude by a factor of 20, which is due to the fact that the Richter magnitude of the earthquake was defined according to the logarithm of maximum amplitude, but AE amplitude was expressed in decibels [52]. Hence, the AE *b_L_*-value was expressed as follows:

(10) $$Log_{10}N\left(\geq A_{dB}\right)=a_{L}-b_{L}\frac{A_{dB}}{20},$$

where *A_dB_* is the amplitude of AE events (dB); *N*(*≥A_dB_*) is the number of events with amplitude greater than *A_dB_* in the region of the first 40 signal points (since the number of AE signal points of asphalt mixture is less than that of concrete or rock); *a_L_* and *b_L_* are the intercept and the negative slope of the regression line in the AE cumulative amplitude distribution based on the least square method.

In addition, the maximum likelihood method based on discrete frequency distribution could be used to calculate the *b*-value, which can be expressed as follows [54]:

(11) $$b_{M}=\frac{20\times \log_{10}\left(e\right)}{\left(\langle A_{dB}\rangle -{A_{dB}}^{*}\right)}$$

where *b_M_* is the AE *b*-value calculated based on the maximum likelihood method; *<A_dB_>* is the arithmetic mean of the amplitude values for a specific window (the first 40 signal points); and A_dB_* is the threshold amplitude (40 dB).

AE energy expresses the relative intensity and severity of cracks and thus can be used to characterize the damage level, because it has been proved to be related to plastic strain energy [55]. Thus, the macro-cracks will excite more AE events with high AE energy, and the micro-cracks will emit more AE events with low AE energy. Referring to Gutenberg’s law, the AE *b_E_*-value was defined as:

(12) $$Log_{10}N\left(\geq E_{A}\right)=a_{E}-b_{E}Log_{10}\left(E_{A}\right)$$

where *E_A_* is the AE energy of signals (mv·ms); *N*(*≥E_A_*) is the number of events with AE energy greater than *E_A_* within the first 40 points; and *b_E_* is the energy *b*-value.

## 3. Results and Discussion

### 3.1. Sentry Function Analysis

**Figure 6:**
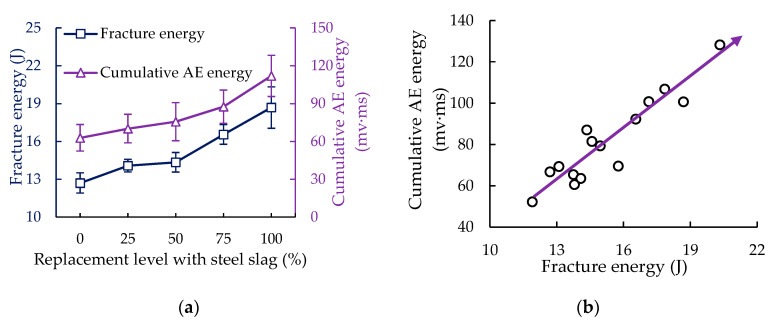
Fracture energy and cumulative AE energy: (**a**) effect of steel slag replacement level on the fracture energy and cumulative AE energy; (**b**) relationship between fracture energy and cumulative AE energy.

The effect of steel slag replacement level on the fracture energy and cumulative AE energy was plotted in Figure 6a. It can be found that the fracture energy increase with the increase of steel slag replacement level, and the low-temperature cracking resistance is improved accordingly. Similarly, the cumulative AE energy also showed an upward trend, and the maximum (111.951 mv·ms) was obtained at the level of 100% steel slag replacement, which is 78.18% higher than that of the control group. It can be found that its increase rate was significantly higher than that of fracture energy (47.16%).

There was a correlation between cumulative AE energy and fracture energy [56]. Figure 6b showed the distribution of cumulative AE energy and fracture energy. It was hard to determine the quantitative relationship between the cumulative AE energy and the fracture energy owing to the differences in the surface roughness of the specimens, the operating methods of the researchers, and the internal structure of the materials [26]. However, from Figure 6b, it could be found that the cumulative AE energy presents an upward trend with the increase of fracture energy in PAM. This qualitative relationship was consistent with the conclusions obtained by Tragazikis et al. and Cortés et al. in the cement mortar and reinforced concrete structures, respectively [25,56].

When the specimen was subjected to an external load, the load work was stored in the form of strain energy inside the materials. From the perspective of energy failure, when the locally stored strain energy exceeded the allowable local strain energy of the materials, the local fractures of material occurred. Then, the instantaneous stress waves could be captured by the AE sensor (piezoelectric transducer) on the surface of the material and then were transformed into electrical signals [32]. As AE energy was the integration along amplitude and time, not the integration along load and displacement in the traditional sense, cumulative AE energy and fracture energy were two different physical concepts, but they presented similar changing trends in low-temperature splitting test.

**Figure 7:**
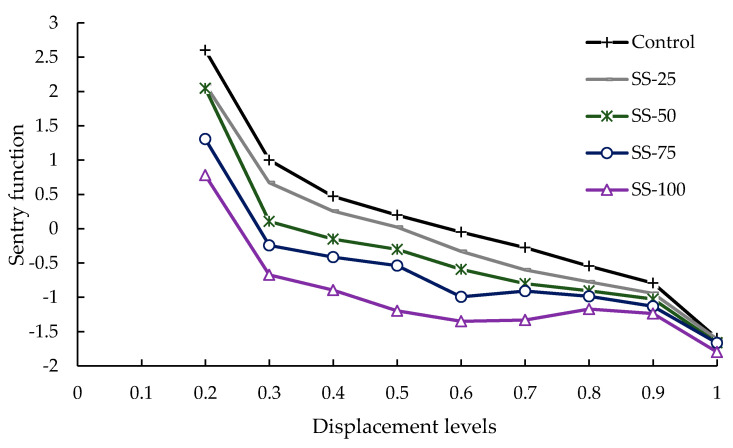
Sentry function of each group at different displacement levels.

The sentry function of each group at different displacement levels was plotted in Figure 7. The obvious downward trend of the sentry function was observed in 0.2–0.3 displacement level for each group, which is associated with the initiation of micro-cracks rather than collapses, as the sentry function in this case is at a relatively high level. Stable decreasing behavior was observed in the 0.3–0.9 displacement level for the control, SS-25 and SS-50 groups, which is associated with the stable propagation of micro-cracks, and this trend was also founded in the 0.6–0.9 displacement level for SS-75 and SS-100. Differently, the near-constant behavior of sentry function was founded in the 0.6–0.9 displacement levels for SS-75, reflecting a balancing phase that the progressive storing of strain energy is equivalent to the steady release of AE energy caused by the crack growth in materials. In particular, a slight bottom-up trend was observed in the 0.6–0.9 displacement level for SS-100, indicating that it has strong strain energy storing capability related to the local resistance and hardening effect generated by the interlocking structure of steel slag coarse aggregates [57]. The sharp drops of sentry function were found in the 0.9–1.0 displacement levels for each group, indicating that the strain energy storing capability of the materials are dramatically deteriorated and the critical load leading to catastrophic failure of the specimen is about to reach.

### 3.2. Rise Angle (RA) and Average Frequency (AF) Analysis

#### 3.2.1. Average RA and AF

**Figure 8:**
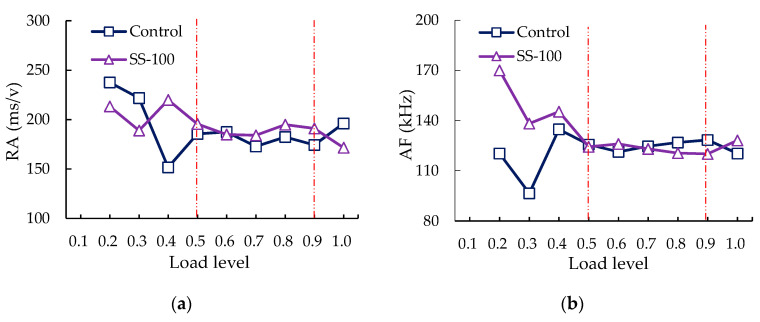
The average fracture mode parameters of the control and SS-100 groups at each load level: (**a**) rise time (RA); (**b**) average frequency (AF).

The average RA and AF at each displacement levels were shown in Figure 8. Only the control group and SS-100 were considered, owing to the SS-100 with the optimal low-temperature cracking resistance and representativeness. From Figure 8a, there was no AE signal below 0.1 load level. Subsequently, RA with large discrete and no obvious trends were observed in the load level from 0.1 to 0.5, both in the control group and SS-100, owing to the lack of signal points in the early stage. Under load levels of 0.5–0.9, the RA of the control group and SS-100 showed a trend of horizontal fluctuation. When the load level was over 0.9, the RA of the control group increased from 174.3 ms/v to 196.2 ms/v and presented a significant upward trend owing to the sharp increase of shear cracks. However, RA of the SS-100 group decreased from 191.0 ms/v to 171.6 ms/v and showed an obvious downward trend.

As can be seen from Figure 8b, the AF below 0.5 load level and between 0.5 and 0.9 load level showed the trend of irregular change and horizontal fluctuation, respectively, which is consistent with the above-mentioned RA trends. When the load level exceeded 0.9, the AF of the control group decreased from 128.5 kHz to 120.4 kHz, and a slight decreasing trend was found. However, the AF of SS-100 increased from 120.1 kHz to 128.2 kHz, and a slightly upward trend was obtained, which runs counter to the trend of RA.

Compared with tensile events, shear events could be distinguished by higher RA and lower AF [58]. Thus, the relative change trend of RA and AF reflected the relative change trend of shear-tensile modes. At the load level exceeding 0.9, a significant drop in RA and a slight increase in AF were obtained in SS-100, but a significant rise in RA and a slight drop in AF were found in the control group, indicating that the shear cracks within the specimens are restricted by the incorporation of steel slag coarse aggregates.

#### 3.2.2. Cumulative RA and AF

**Figure 9:**
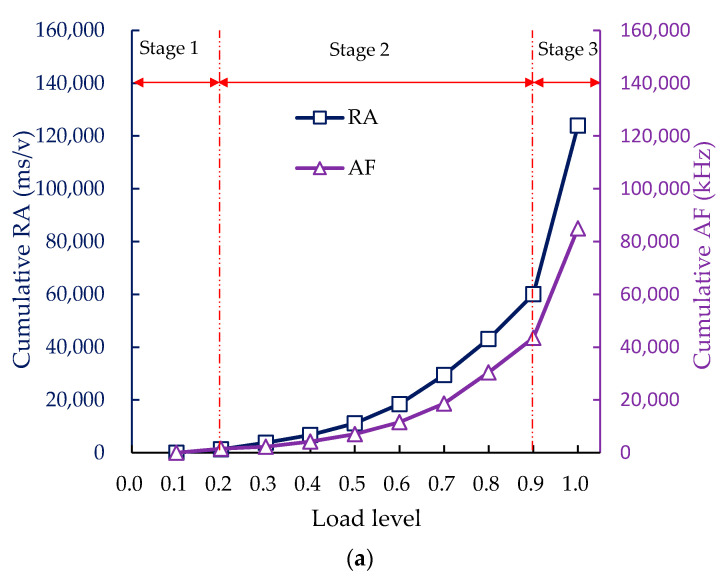

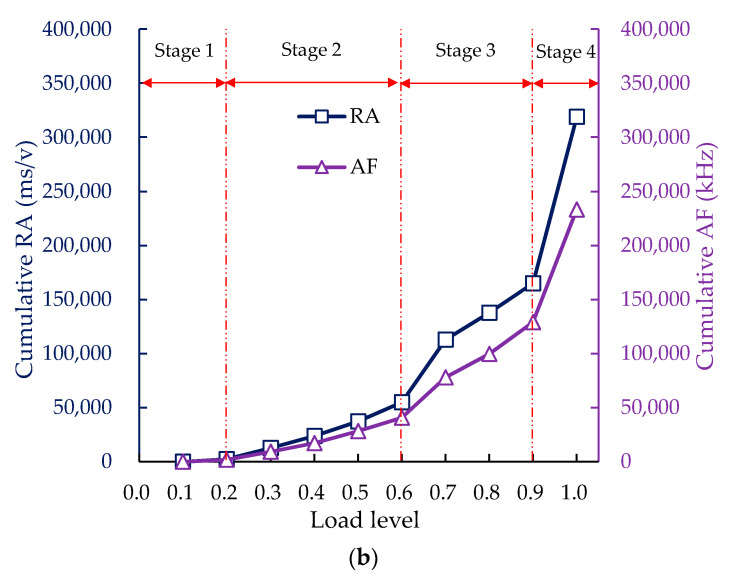
The cumulative RA and cumulative AF at each load level: (**a**) control group; (**b**) SS-100.

The variation of cumulative RA and AF values vs. load levels in low-temperature splitting test was presented in Figure 9. From Figure 9a, the damage evolution process of the control group was divided into three stages based on the slope changes of the cumulative RA and cumulative AF curves. A slight change, steady growth, and abrupt surge of cumulative RA and cumulative AF curves were observed in the compaction stage (before 0.2 load level), micro-crack stage (between 0.2–0.9 load level), and macro-crack stage (over 0.9 load level), respectively [58].

From Figure 9b, a near-horizontal trend, linearly increasing trend, and sharp increase were also found in the compaction stage (before 0.2 load level), micro-crack stage (load levels of 0.2–0.9), and macro-crack stage (upper 0.9 load level) of the SS-100, respectively, which is similar to the trend found in the control group. However, a local mutation of the cumulative RA and cumulative AF was found at the 0.6–0.7 load levels, and then a gentle growth was observed at the 0.7–0.9 load levels. Thus, the phase between 0.6 and 0.9 load levels was considered as the local resistance stage, since the above-mentioned gentle growth correlates with the local resistance generated by the interlocking structure of steel slag coarse aggregates. The existing paper has proved that the interlocking structure is related to the multi-angularity and cubic shape of steel slag coarse aggregate [17]. The phase division based on cumulative RA and cumulative AF was similar to that based on AE energy reported in the author’s previous research, proving that cumulative RA and cumulative AF can also realize the identification of the damage stages in the low-temperature splitting test [57,58].

#### 3.2.3. The Determination of the Boundary Line

**Figure 10:**
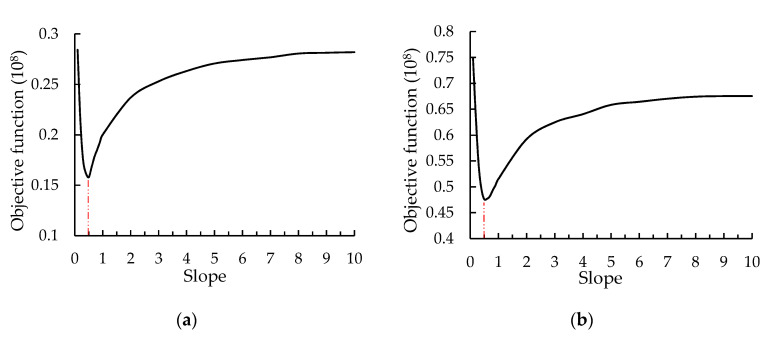
The relationship between the objective function and the slope: (**a**) control group; (**b**) SS-100.

The change of the objective function vs. the slope of the boundary line was depicted in Figure 10. From Figure 10, the objective functions of the control group and SS-100 showed a similar trend, that a significant drop before the slope of 0.5 and a bottom-up trend after exceeding the slope of 0.5. Therefore, the slope of the decisive boundary line was selected as 0.5 based on the minimum dissimilarity of clustering—that was, the objective function reached the minimum value. Thus, the boundary line was determined as AF/RA = 0.5.

#### 3.2.4. Fracture Mode Classification

**Figure 11:**
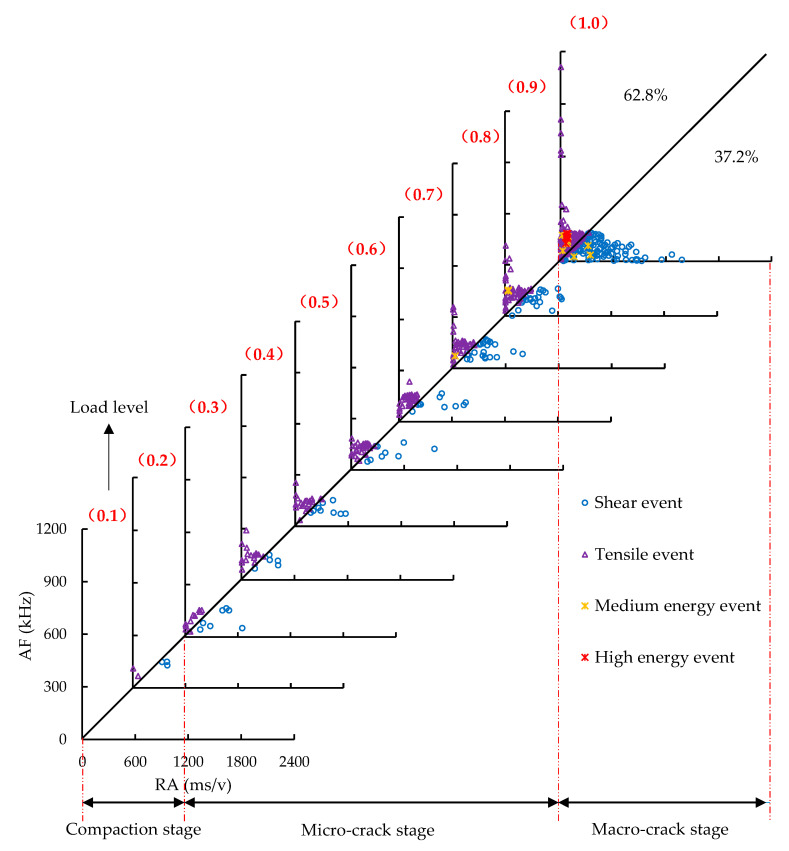
The distribution of RA and AF at each load level in the control group.

The distribution of RA and AF at each load level in the control group was shown in Figure 11. At the compaction stage, a small number of signal points with low RA and AF were observed, indicating that the damage of the specimen was relatively low. These signals were mainly generated by the internal void compaction of the specimen. In the micro-crack stage, more AE signals and larger RA and AF were found in comparison to the compaction stage, which is due to the nucleation and stable propagation of micro-cracks. In the macro-crack stage, dense signal points were found, especially for those with RA within 600–1200 ms/v, indicating that a large number of shear fractures occur, which is involved in the formation of macro-cracks generated by the extended and merged micro-cracks. A small number of events with medium and high AE energy appeared, indicating that the bearing capacity of specimens is about to reach the critical state and the specimen will be destroyed due to the unstable propagation and coalescence of macro-cracks.

**Figure 12:**
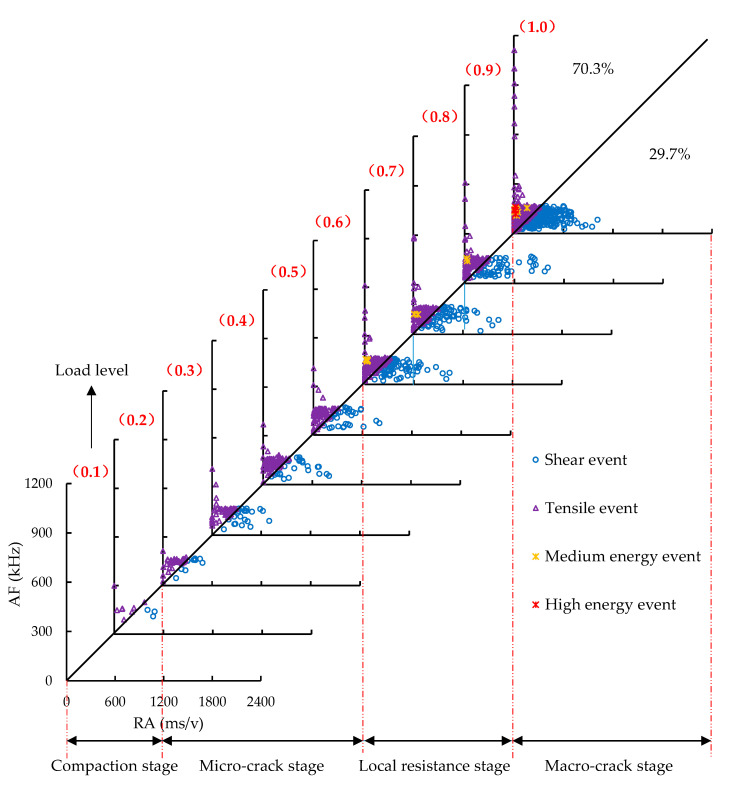
The distribution of RA and AF at each load level in the SS–100.

The distribution of RA and AF at each load level in the SS-100 was presented in Figure 12. It could be found that similar phenomena appeared in the compaction, micro-cracks, and macro-cracks stages of the SS-100. Differently, there were fewer events with high RA (over 700 ms/v), and the shear events were reduced from 37.2% to 29.7% in the macro-crack stage after adding steel slag, showing that the growth of interfacial shear fracture in the specimen is restrained. The reasons lied in the strong adhesion between the steel slag aggregates and bitumen. The strong adhesion was associated with the peculiar porous characteristics, rough texture, and alkalinity of the steel slag [59]. Furthermore, it can be found that the middle- and high-energy events of the SS-100 were mainly centralized in the tensile mode, because the AE energy of tensile crack is substantially higher than that of shear crack [25]. In the control group, not only those events with a greater number concentrated in the tensile mode were found, but also the medium energy events with a small number were observed in shear cracks. AE energy was associated with crack intensity and the crack intensity of the SS-100 was relatively low. Under a high load level, the macro-cracks of basalt aggregates occurred with shear fractures, thus resulting in the loss of aggregate supporting capacity and the occurrence of more macro-cracks at the interfaces between aggregates and binders with the tensile mode. The above phenomenon was not significant in the SS-100, owing to the superior strength and interlocking structure of the steel slag aggregates. In the local resistance stage, the shear cracks with RA from 600 ms/v to 1200 ms/v increased obviously, which is related to the dislocation movement of steel slag coarse aggregates. The dislocation movement forms the subsequent interlocking structure.

### 3.3. b-Value Analysis

**Figure 13:**
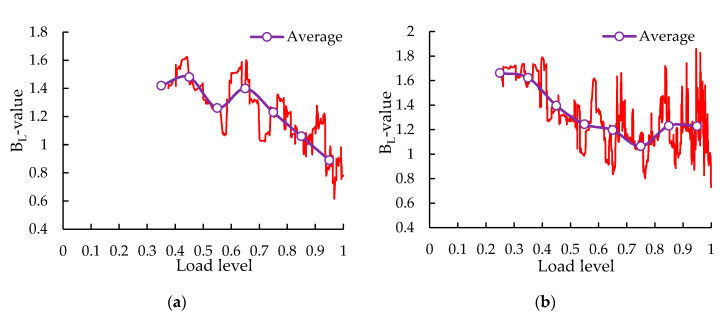
The *b_L_*-values at each load level: (**a**) control group; (**b**) SS-100 group.

The *b*-values could not be calculated and were not found before the low-level load because *b*-values were obtained by the amplitude of the first 40 signal points and the signal points were less in the early stage. The *b*-value and average *b*-value were described by the red line and purple line, respectively. The *b_L_*-values at each load level were presented in Figure 13. Through the red line, it could be found that the *b_L_*-value is fluctuant at each load level, rather than constant, monotonously increasing or decreasing, which is associated with the heterogeneous texture of specimens and the coupling phenomenon of micro-cracks and macro-cracks [60]. As can be seen from the average line, *b_L_*-values presented an overall downward trend, although there were fluctuations in local regions, indicating the progressive damage evolution of the specimens. The reasons lied in that the nucleation and stable growth of micro-cracks in the early stage produce a large number of events with low amplitude, resulting in the high *b_L_*-value, and the formation and unstable propagation of macro-cracks excite a small number of events with high amplitude, leading to the low *b_L_*-value. From Figure 13a, it could be found that the *b_L_*-value abruptly decreases from 1.28 to 0.62 in the final stage for the control group without significant repeated fluctuation, indicating that the fracture modes shifted from micro-cracks to macro-cracks, and then macro-cracks rapidly merged into critical macro-cracks, leading to brittle failure and collapse of the specimen [60]. This brittle failure was mainly attributed to the brittle fractures of basalt aggregates under the high load level (over 0.9 load level). The weakening of the support force from basalt aggregates induced the unstable propagation and rapid combination of the macro-cracks at the interfaces between aggregates and binders. From Figure 13b, the phenomena of the *b_L_*-value with the significant repeated fluctuation (from 1.86 to 0.73) and a not-obvious downward trend were observed in the final stage for the SS-100, which is different from those found in the control group. The significant repeated fluctuation showed that the coupling phenomenon of micro-cracks and macro-cracks intensified and the specimen was about to be destroyed. In addition, the large fluctuation accompanied by a not-obvious drop was related to the ductile failure of the SS-100. There were several reasons that accounted for it. On the one hand, the high strength and interlocking structure of the steel slag aggregates restrained the development of the macro-cracks in aggregates and binders, which weaken the phenomenon of brittle fracture of aggregates [59]. On the other hand, the rough and porous texture of the steel slag aggregates could absorb more structural bitumen [61]. Furthermore, the existing studies showed that the chemical acid–base reaction between weak acid bitumen and strong basic steel slag enhances the adhesion between aggregate and bitumen [62,63]. All of those were conducive to enhancing the ductility of the SS-100. The sharp drop and significant fluctuation of *b_L_*-values could be known as serious damage alerts of the control group and SS-100, respectively.

**Figure 14:**
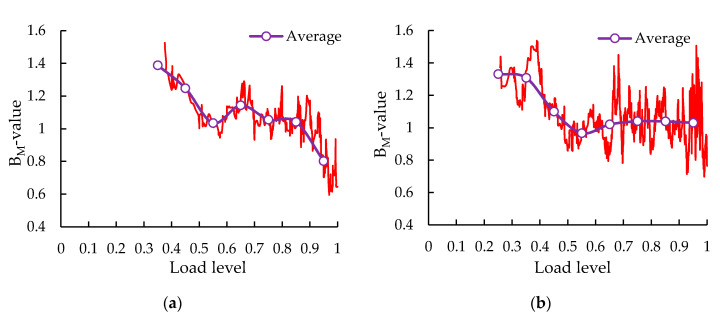
The *b_M_*-values at each load level: (**a**) control group; (**b**) SS-100 group.

The *b_M_*-values at each load level were shown in Figure 14. It can be found that both methods present similar trends: the sudden drop of the *b_M_*-value of the control group and the distinct repeated fluctuation of the *b_M_*-value of the SS-100 in the final stage. Compared with the least square method, the *b_M_*-value calculated by the maximum likelihood method was simpler, and that of the control group in the final stage decreased more significantly, owing its high sensitivity to high-amplitude events.

**Figure 15:**
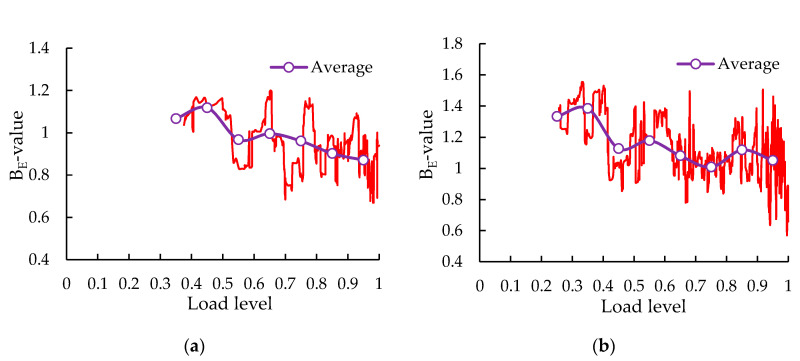
The *b_E_*-values at each load level: (**a**) control group; (**b**) SS-100 group.

AE energy yielded a more robust evaluation of the cracking severity, due to the fact that it is the area under the rectified signal envelope and contains more information about the signal source. Thus, it was less susceptible to the attenuation and distortion caused by reflections, transmission, and scattering of elastic waves, in comparison with amplitude [55]. The *b_E_*-values at each load level are shown in Figure 15. The above-mentioned phenomenon of amplitude *b*-value was also found in *b_E_*-value, indicating that the test results obtained by AE monitoring are reliable. The *b_E_*-value showed a downward trend on the whole, indicating that a large number of signals with low energy appear in the early stage and a small number of signals with medium and high energy occur in the final stage, which is related to the gradually increasing intensity of cracks.

## 4. Conclusions

In this study, the low-temperature fracture behavior of PAM with steel slag was monitored in real time by employing the AE technique. The sentry function, RA, AF, and *b*-values were used as indexes to evaluate the fracture behavior. Based on the test results, the following conclusions could be drawn:

1. The cumulative AE energy increased with the increase of steel slag replacement level. A slight bottom-up trend of sentinel function was observed in the 0.6–0.9 displacement level for SS-100, which is associated with an enhanced strain energy storing capability generated by the interlocking structure of steel slag coarse aggregates.

2. The fracture stages of the control group and SS-100 could be divided based on the slope changes of the cumulative RA and cumulative AF curves. The incorporation of 100% steel slag reduced the shear events and restrained the growth of shear cracking of the specimen in the macro-crack stage.

3. The considerable drops of three kinds of *b*-values in the final phase were registered in the control group, but there were significant repeated fluctuations in SS-100, which are associated with the brittle failure of the control group and the ductile failure of the steel slag group, respectively. The sharp drops and significant fluctuations of *b*-values could serve as a serious damage alert of the control group and SS-100, respectively.

4. The fracture behavior of PAM was improved significantly after adding 100% steel slag. AE technique possessed great potential for real-time characterizing of fracture behavior of PAM containing steel slag and was expected to be further applied in pavement engineering.

## Figures and Tables

**Table 1:** Properties of SBS-modified bitumen.

Properties	Test Values	Specification
Penetration (25 °C, 0.1 mm)	69.2	60–80
Softening point (°C)	63.5	≥55
Ductility (5 °C, cm)	42.7	≥30
Flash point (°C)	261	≥230
Elastic recovery (25 °C, %)	91.6	≥65
After TFOT		
Mass loss (%)	0.24	≤±1.0
Penetration ratio (25 °C, %)	67	≥60
Ductility (5 °C, cm)	31	≥20

**Table 2:** Properties of coarse aggregates.

Item	Basalt	Steel Slag	Specification
Los Angeles abrasion (%)	17.9	12.7	≤28
Flakiness content (%)	4.8	4.53	≤10
Crushed value (%)	16.8	13.9	≤26
Expansion (%)	0.12	0.44	≤2.0

**Table 3:** Properties of fine basalt aggregates.

Item	Test Values	Specification
Apparent density (g/cm^3^)	2.64	≥2.5
Water absorption (%)	1.13	—
Angularity (s)	39.5	≥30
Sand equivalent (%)	71.4	≥60

**Table 4:** The experimental gradation of each group.

Sieve Size (mm)	Control	SS-25	SS-50	SS-75	SS-100
0.075	5.0	4.8	4.6	4.4	4.3
0.15	6.8	6.5	6.3	6.0	5.8
0.3	7.9	7.6	7.3	7.0	6.8
0.6	10.5	10.1	9.7	9.3	9.0
1.18	12.9	12.4	11.9	11.5	11.1
2.36	16.7	16.0	15.4	14.8	14.3
4.75	23.4	23.0	22.6	22.2	21.8
9.5	66.4	66.2	66.0	65.8	65.7
13.2	94.7	94.7	94.7	94.7	94.7
16	100.0	100.0	100.0	100.0	100.0

**Table 5:** Volume characteristics and mechanical properties test results.

Items	Control	SS-25	SS-50	SS-75	SS-100	Specification
VV ^1^ (%)	19.89	20.78	21.6	21.8	21.81	18–25
VMA ^2^ (%)	26.49	27.11	27.70	27.93	27.81	—
VFA ^3^ (%)	24.90	23.35	22.01	21.96	21.56	—
Permeability (mm/s)	2.85	2.97	3.12	3.23	3.25	≥2.80
Cantabro abrasion loss (%)	12.8	11.2	10.6	9.5	8.7	≤15
Draindown (%)	0.28	0.27	0.25	0.24	0.21	≤0.3
MS ^4^ (kN)	6.75	7.59	7.64	8.21	8.51	≥5.0
FL ^5^ (0.1mm)	31.5	32.2	32.6	32.5	31.8	20–40
ITS ^6^ (MPa)	0.633	0.635	0.659	0.706	0.749	—
TSR ^7^ (%)	85.63	89.34	89.37	90.65	90.85	≥85%

^1^ Volume of air voids. ^2^ Voids in mineral aggregate. ^3^ Voids filled with asphalt. ^4^ Marshall stability. ^5^ Flow value. ^6^ Indirect tensile strength. ^7^ Tensile strength ratio.

**Table 6:** Low-temperature splitting test results.

Items	Control	SS-25	SS-50	SS-75	SS-100
LITS ^1^ (MPa)	2.977	2.972	2.940	2.766	2.727
Failure strain (10^−6^)	2307	2544	2626	3204	3946
Deformation energy (J)	12.70	14.08	14.35	16.55	18.69

^1^ Low-temperature indirect tensile strength.

**Table 7:** The corresponding standard deviation of test results.

Items	Control	SS-25	SS-50	SS-75	SS-100
VV (%)	0.33	0.36	0.35	0.37	0.39
VMA (%)	0.16	0.16	0.18	0.19	0.19
VFA (%)	0.19	0.25	0.20	0.26	0.33
Permeability (mm/s)	0.04	0.06	0.05	0.05	0.06
Cantabro abrasion loss (%)	0.20	0.21	0.20	0.23	0.22
Draindown (%)	0.01	0.01	0.01	0.01	0.02
MS (kN)	0.28	0.30	0.30	0.33	0.34
FL (0.1 mm)	0.16	0.21	0.20	0.24	0.21
ITS (MPa)	0.02	0.01	0.01	0.01	0.02
TSR (%)	0.41	0.45	0.51	0.48	0.51
LITS (MPa)	0.02	0.02	0.03	0.02	0.03
Failure strain (10^−6^)	146.21	131.62	184.25	251.84	205.22
Deformation energy (J)	0.61	0.33	0.42	0.58	0.63
